# Cytosolic Ascorbate Peroxidases Plays a Critical Role in Photosynthesis by Modulating Reactive Oxygen Species Level in Stomatal Guard Cell

**DOI:** 10.3389/fpls.2020.00446

**Published:** 2020-05-07

**Authors:** Kai Guo, Zhonghua Li, Hanxue Tian, Xueqiong Du, Zhen Liu, Hui Huang, Pengcheng Wang, Zhengxiu Ye, Xianlong Zhang, Lili Tu

**Affiliations:** ^1^National Key Laboratory of Crop Genetic Improvement, Huazhong Agricultural University, Wuhan, China; ^2^College of Agronomy and Biotechnology, Southwest University, Chongqing, China

**Keywords:** cotton yield, cytosolic ascorbate peroxidases, photosynthesis, reactive oxygen species, stomata

## Abstract

Photosynthetic rate is one of the key factors limiting yield of cotton. Reactive oxygen species (ROS) generated by abiotic stress imposes numerous detrimental effects and causes tremendous loss of yield. It is worth to study whether ROS scavenging enzymes could affect yield through regulating photosynthetic rate in cotton. In this study, we created transgenic cotton with changes of endogenous ROS by overexpressing or suppressing the expression of cytosolic ascorbate peroxidases (*APX*s), which are hydrogen peroxide (H_2_O_2_) scavenging enzymes in plants. The suppression of cytosolic *APX*s by RNAi brings about a great influence on plant growth and development. Plant height and leaf size declined, and yield-related traits including single boll weight, seed weight, seed size, and lint weight dropped significantly, in IAO lines (cytosolic *APX*-suppressed lines). The stunted plant growth was due to the decrease of plant photosynthetic rate. The evidences showed that increased ROS level in guard cells inhibited stomatal opening and suppressed the absorption of CO_2_ and H_2_O in IAO line. The decrease of water content and the increase of water loss rate in leaf exacerbated the decline of photosynthetic rate in cytosolic *APX*-suppressed lines. Based on these results, it implies that cytosolic *APX*s as a whole play an important role in maintaining REDOX balance to regulate photosynthetic rate and yield in cotton.

## Introduction

Photosynthesis refers to the process in which leaves use light energy to change the absorbed carbon dioxide and water into sugar and release oxygen in green plants. It is the primary producer of organic matter and energy on earth. Photosynthetic efficiency plays a critical role in crop production and is an important factor limiting crop yield (Zhu et al., 2010). Most of dry matter in crops, 90 to 95%, is produced by photosynthesis through carbon assimilation. Therefore, high photosynthetic efficiency is an important indicator of high yield in crops (Evans, 2013). At present, the light energy utilization rate is still very low, generally only 1–2% in crops. It is estimated that if the light energy utilization rate increased to 2.4–2.6%, the yield can reach to 1,000 kg per unit of acres in wheat and rice (Eva et al., 2019; Simkin et al., 2019). Therefore, there is great potential to improve the photosynthetic efficiency for high yield production in crops.

External factors light intensity, light quality, carbon dioxide concentration, water, and internal factors leaf physiological state and structure are the key factors that affect photosynthetic efficiency in crops (Zhu et al., 2010; Evans, 2013). These factors either regulate the supply of assimilates in source organs such as functional leaves or germinating cotyledon or control the accumulating rate of assimilates in sink organs like fruits or seeds. Therefore, reactive oxygen species (ROS) generated by adverse environmental stresses, such as drought, heat, heavy metal toxicity, and high light, could significantly reduce photosynthetic efficiency (Gururani et al., 2015; Smirnoff and Arnaud, 2019). As the by-product of photosynthesis, ROS is mainly generated in chloroplasts within the electron transport chains of photosystem II (PSII) and photosystem I (PSI) during light reactions (Foyer and Noctor, 2013). The main detrimental effects of ROS accumulation in chloroplasts are to destroy the balance of photosynthetic REDOX system, cause photoinhibition, and inhibit the damage repair of PSII (Gururani et al., 2015), including the inhibition of *de novo* synthesis of D1 protein, which is needed for PSII repair (Nishiyama et al., 2011; Tikkanen et al., 2011; Gururani et al., 2015), suppression of ROS-responsive chloroplast enzymes (Yoshioka et al., 2006; Yoshioka-Nishimura et al., 2014), and the disarrangement of thylakoid architecture (Shen, 2015). Under stress condition, the absorption of carbon dioxide in leaves would be decreased, which will further promote the accumulation of ROS, causing great damage to photosynthetic organs, especially PSII (Gururani et al., 2015).

Recent studies in various plants suggest that ROS also plays a pivotal role as a signaling molecule in biochemical and physiological responses including hormone interaction network, MAPK cascade, Ca^2+^ signaling network, and transcription factor regulation pathway (Apel and Hirt, 2004; Considine and Foyer, 2014). The opening and closing of stomata are the major way to control water transpiration and the diffusion of gases into and out of air spaces in plants. Stomatal guard cells are the main target of ROS signaling, depending on ABA and non-ABA signaling pathway to regulate ion changes to control plant response and development (Wang and Song, 2008; Song et al., 2014). The increased ROS can induce the internal flow of calcium ions in guard cells to increase the concentration of cytosolic free calcium ([Ca^2+^]_*cyt*_), inhibit stomatal opening, and cause stomatal closure (Pei et al., 2000). ROS can regulate the contents of potassium, acetate, and malate in guard cells; change the turgor pressure of cells; and affect the pore size of stomatal aperture (Dong et al., 2018). ROS can also modify phosphorylation sites of some important proteins in guard cells to promote the formation of protein disulfide bonds and change the conformation of proteins to regulate the size of stomata (Miao et al., 2006; Wang et al., 2010). It is also well-understood that guard cells are capable of adjusting stomatal aperture in response to multiple biotic and abiotic stresses, which balance the loss of water and the absorption of carbon dioxide to support photosynthesis for plant growth and development (Song et al., 2014). Therefore, guard cells are widely regarded as important conversion stations to maximize water use efficiency and carbon dioxide exchange rate to maintain photosynthesis.

To overcome ROS generation by adverse environmental stresses, a series of reducing chemicals [like ascorbic acid (ASA) and glutathione (GSH)] and ROS scavenging enzymes [such as ascorbate peroxidase (APX), catalase (CAT), peroxidase (POD), and GSH peroxidase (GPX)] were produced to maintain the balance of intracellular ROS level and the stability of intracellular redox state (Smirnoff and Arnaud, 2019). APX is a plant-specific heme-containing peroxidase balancing the cellular redox state for normal plant growth through utilizing reduced ASA as its specific electron donor to reduce H_2_O_2_ to H_2_O, with the concomitant generation of dehydroascorbic acid (DHA) (Foyer and Noctor, 2013).

According to the location in cell, *APX* can be divided into four types including cytosolic *APX* (*cAPX*), chloroplastic *APX* (*chlAPX*), mitochondrial *APX* (*mitAPX*), and peroxisomal *APX* (*mAPX*) (Pandey et al., 2017). Until now, there were eight *APX* genes founded in rice and *Arabidopsis* genome, and each type of *APX* has two genes. The cytosolic type of *APX* has two genes, *APX1* and *APX2*, and plays a critical role in plant development by maintaining redox homeostasis (Bonifacio et al., 2011; Suzuki et al., 2012; Begara-Morales et al., 2013). *cAPX* genes can be induced to protect photosynthesis under a variety of biotic and abiotic stresses in plant, including high light, high temperature, drought, salinity and alkali stress, and mineral element deficiency (Pandey et al., 2017). Photosynthetic electron transport chain system under high light stress could induce the expression of *cAPX* genes to overcome the burst of ROS production, which releases the inhibition of photosynthesis caused by the explosion of H_2_O_2_ photooxidation (Karpinski et al., 1997; Mittler et al., 1998). In *Arabidopsis*, cytosolic *atapx1* mutants showed high sensitivity to the oxidative damage induced by MV (Methyl Viologen), HL (High Light), and drought (Rizhsky et al., 2002; Pnueli et al., 2003; Chang et al., 2004; Davletova et al., 2005; Lu et al., 2007; Miller et al., 2007; Koussevitzky et al., 2008; Suzuki et al., 2012). The *cAPX2* gene can also be induced quickly by the high light and high temperature stress to protect the damage caused by light oxidative stress in plant leaves. Loss function of *OsAPX2* affected the growth and development in rice seedlings by protecting the seedlings from abiotic stresses (Zhang et al., 2013; Wu et al., 2018). Therefore, *cAPX*s plays an important role in balancing plant growth and resistance to oxidative stress. It is just that there are differences in the function of cytosolic type of gene in different plants.

In our previous researches, we found that the suppression of *cAPX*s increased tolerance to Fe deficiency by modulating ABA level in cotton, and the specific suppression of *GhAPX1* was unable to alter the response of cotton to Fe deficiency (Guo et al., 2016b). Moreover, *cAPX*-suppressed cotton fibers showed more sensitivity to oxidative stress than did wild-type plants, and the overexpression of *GhAPX1* improved the tolerance of fibers to oxidative stress in cotton (Guo et al., 2016a). *cAPX*s in cotton displayed a function different from that in rice and *Arabidopsis*.

In this paper, we explored the role of *cAPX*s in protecting cellular oxidative homeostasis of stomata guard cells and the function to maintain photosynthesis in cotton. *GhAPX1* overexpressed and *cAPX* suppressed by RNAi transgenic cottons were constructed through *Agrobacterium*-mediated transgenic technology. The content of H_2_O_2_ in the transgenic cotton cells changed, and the plant morphology, leaves, seed, and fiber were different from those of wild-type plants. Further research showed that the increase of endogenous ROS level in guard cells inhibited stomatal opening, increased the water loss rate of leaves, led to the decrease of carbon dioxide fixation and water content, and finally significantly decreased net photosynthetic rate of plants and decreased the yield of cotton.

## Materials and Methods

### Materials

Transgenic cotton plants were created in the previous research (Guo et al., 2016a). The open reading frame (ORF) sequence of *GhAPX1* (Gh_A05G0863) cDNA was amplified and inserted into the 35S overexpression vector pK2GW7.0 (Ghent University)^1^ to construct *GhAPX1*-overexpressed lines OA15 and OA17. The ORF of *GhAPX1* was inserted into the plasmid pHellsgate4 to create *cAPX* suppressed lines IAO24 and IAO167. All transgenic cottons and the control plants were grown in the experimental field at Huazhong Agricultural University in Wuhan, Hubei Province, PR China.

### RNA Extraction and RT-qPCR

All samples of transgenic cottons for RNA extraction were collected at 8 o’clock in the morning and 8 o’clock in the evening, respectively. The leaves were collected in liquid and ground into powder. Total RNA was extracted according to a previously described method (Guo et al., 2016a), and cDNA was synthesized with M-MLV Reverse Transcriptase (Promega, Madison, WI, United States) according to the manufacturer’s instructions. RT-qPCR was performed with fluorescent dye SYRB (BIO-RAD) as previously described using an Applied Biosystems 7500 Real-Time PCR System (Guo et al., 2016a). *GhUB7* (DQ116411) served as the internal control to normalize expression levels. Primers used for the detection of *cAPX*s in the study are listed in Supplementary Table 1.

### Detection of Photosynthetic Rate in Transgenic Plants

At full bloom stage, the photosynthesis of the third upper leaves of the transgenic and control cotton was measured at about 10 o’clock in the morning on a clear day. The portable field photosynthetic rate meter IRGA LI-6400XT (LI-COR, Lincoln, NE, United States) was used for measurement with a portable infrared gas analyzer (IRGA) system, equipped with a LED source and a leaf chamber (2 cm × 3 cm) (Bonifacio et al., 2011). The internal parameters in the IRGA chamber during gas exchange measurements were as follows: 1,500 μmol⋅m^–2^s^–1^ of photosynthetic photon flux density (PPFD), 1.0 ± 0.2 kPa of vapor pressure deficit (VPD), 38 Pa of CO_2_, and temperature of 32°C. Each one of these conditions was separately controlled in the IRGA leaf chamber. The standard leaf compartment was used to simulate natural light. The fixed light intensity was measured at 1,500 mol⋅m^–2^s^–1^, and the main photosynthetic physiological indexes of the third upper leaf were measured. At least six plants per transgenic line were detected.

### Initiated Fiber Number Detection Using Scanning Electron Microscopy

For the detection of initiated fiber number, ovules were collected on the morning of flowering day [0 day post anthesis (DPA)] at full bloom stage. The flowers at the same branch and node in the middle of transgenic cottons were selected. Three flowers for each plant line and eight ovules of the same part in one ovary were selected for ovules and fixed in solution [2.5% (v/v) glutaraldehyde phosphate buffer (pH 7.2)]. After that, 30, 50, 70, 85, 95, and 100% (two times) ethanol was gradually used for gradient dehydration, 15 min for each grade, with appropriate shaking to ensure enough dehydration. Then iso-amyl acetate:ethanol = 1:1 (v/v) mixture and iso-amyl acetate were soaked for 10 min, respectively, for substitution. Finally, enough drying is carried out on the critical point of CO_2_ drying. After the fully dried adaxial surface of the leaf was pasted on the sample table with conductive adhesive and the coating process on the abaxial surface of the leaf with ion sputtering apparatus was finished, observation can be made with JSM-6390/LV scanning electron microscope (JEOL, Tokyo, Japan). The image with a magnification of 600 times was used for calculating the number of initial fibers.

### Reactive Oxygen Species Fluorescence Detection in Stomatal Guard Cells

Reactive oxygen species detection in stomatal guard cells was performed using the fluorescent indicator dye 2′,7′-dichlorodihydrofluorescein diacetate (2′,7′-DCFDA; D6883, Sigma-Aldrich, United States) according to a previously reported method (Pei et al., 2000). The transgenic and control plants were at full bloom stage, and the third upper leaves were collected. The epidermal cells of abaxial leaves were removed with tweezers and placed in a phosphate buffer containing 10 μM of 2′,7′-dichlorotoluene yellow acetic acid [phosphate-buffered saline (PBS)] (0.01M of PBS: 135 mM of NaCl, 2.7 mM of KCl, 1.5 mM of KH_2_PO_4_, and 8 mM of K_2_HPO_4_). Incubation was 30°C for 30 min under dark conditions. Then they were cleaned with sterile water and set aside for 5 min. Then they were placed carefully on the slide, and a drop of PBS was added to cover the slide. Fluorescence observation and photography were performed under Laser TCS SP2 confocal microscope. The excitation wavelength of the dye is 488 nm, and the emission wavelength is 522 nm. At least five leaves were observed for each line.

### The Measure of Water Content and Water Loss Rate of Leaves

The third upper leaves of the transgenic and control plants were used to detect water content and water loss rate of leaves. For leaf water content, the third upper leaves of transgenic plants and the control were collected and placed on ice, brought back to the lab to weigh the fresh weight, and then oven-dried to the constant weight at 80°C. The value of fresh weight minus dry weight was divided by fresh weight and then multiplied by 100% to obtain the water content for each leaf. There were four biological repeats for each line. At full bloom stage, the third upper leaves of transgenic and control plants were collected, placed on ice, and taken to the lab. After full absorption of water to the constant weight, the leaves were placed on a filter paper in a relatively closed environment at room temperature, where leaf weight was measured every 20 min and recorded until the leaf weight did not change significantly. The water loss rate was calculated by subtracting the fresh weight of the leaves from the fresh weight of the water absorption at a certain time point, dividing by the fresh weight of the leaves, and then multiplying by 100%, which was the water loss rate at the corresponding time point. There were four biological repeats for each line.

### The Measurement of Glucose, Fructose, and Sucrose

Cotton bolls were collected at 10 DPA in the morning (AM) and afternoon (PM). Fibers were removed from the ovules in liquid nitrogen and ground to a powder. Samples (0.07 g) were extracted in a 10-ml tube with 6 ml of 80% ethanol, heated to 80°C for 30 min, and then centrifuged at 12,000 × *g* for 15 min. The supernatant was used for the assay of glucose, fructose, and sucrose according to a previous method (Guo et al., 2016b).

### The Measurement of Yield-Related Traits in Cotton

Cotton bolls from the middle of transgenic and control plants were taken, and the weight of a single boll was weighed, namely, the weight of a single boll, and 20 ripe bolls for each line were checked. Fibers and seeds of 100 fiber-bearing seeds were separated. The weight of 100 seeds is the seed weight, and the weight of fiber is the lint weight. The length and width of seeds were measured using a vernier caliper. At least 20 seeds were measured for each line.

## Results

### The Downregulation of Cytosolic Ascorbate Peroxidases Suppressed the Growth and Development of Source Organs in Cotton

**FIGURE 1:**
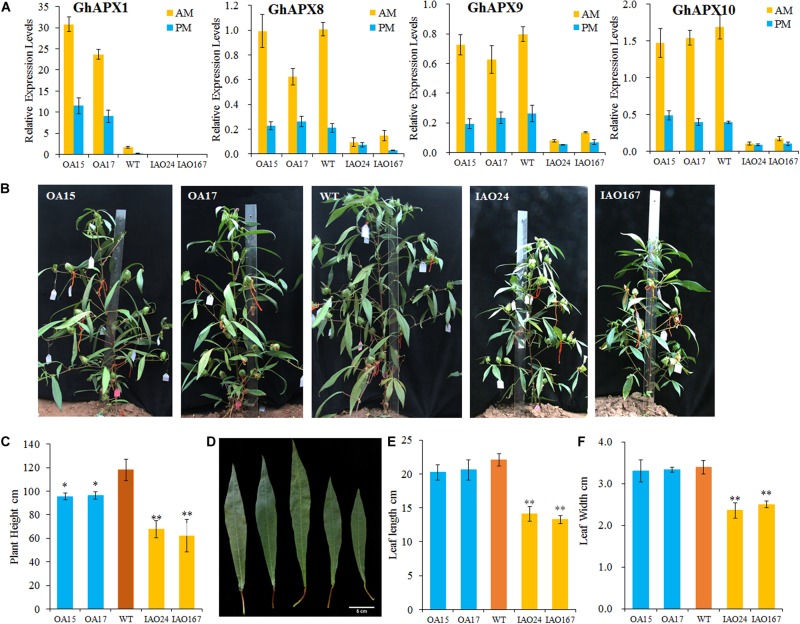
The development of source organs was suppressed in cytosolic ascorbate peroxidase (*APX*) interfered cotton. **(A)** Relative expression levels of cytosolic *APX*s compared with the reference gene *GhUB7* in transgenic cotton. OA, 35S-overexpressed *GhAPX1* lines; WT, wild-type cotton; IAO, the interference of cytosolic *APX*s transgenic lines. *GhUB7* used as the reference gene for the relative expression level. Three biological duplicated experiments were performed. AM and PM indicated that leaves for expression level detection were collected in the morning and afternoon, respectively. **(B)** Plant architecture of *GhAPX1*-overexpressed plants and cytosolic *APX*-suppressed plants compared with the wild-type plants. **(C)** The plant height of transgenic cotton plant in field with three independent experiments (mean ± SD, *n* = 8 plants of each line). **(D)** The third upper leaf of *GhAPX1*-overexpressed and cytosolic *APX*-suppressed plants. **(E)** The leaf length of the third upper leaf in transgenic plant with three independent experiments (mean ± SD, *n* = 8 plants of each line). **(F)** The leaf width of the third upper leaf in transgenic plant with three independent experiments (mean ± SD, *n* = 8 plants of each line). ^∗^Indicates significant difference using Duncan’s multiple comparisons (^∗^*P* < 0.05; ^∗∗^*P* < 0.01).

To check the role of *cAPX*s in photosynthesis, we created *GhAPX1* 35S-overexpressed transgenic cottons and *cAPX*-suppressed cottons. The expression levels of *cAPX*s were confirmed by RT-qPCR in leaves collected from transgenic plants in the morning (AM) and afternoon (PM) (Figure 1A). The expression level of *GhAPX1* was upregulated in the 35S-overexpressed lines OA15 and OA17 and downregulated in *cAPX*-suppressed lines IAO24 and IAO167 (Figure 1A). The expression levels of other *cAPX* members, *GhAPX8*, *GhAPX9*, and *GhAPX10*, were not upregulated in OA15 and OA17 lines, but all were suppressed in IAO24 and IAO167 (Figure 1A). These results suggested that *GhAPX1* was upregulated in OA15 and OA17 and that *cAPX*s were suppressed in IAO24 and IAO167. The expression pattern of *cAPX*s in transgenic cotton levers was consistent with that in fibers and ovules (Guo et al., 2016a).

We found that the plant growth and plant size were seriously inhibited in the *cAPX*-suppressed cottons (Figure 1B). The plant heights were significantly decreased in IAO24 line (67.56 ± 7.25 cm) and IAO167 line (62.13 ± 13.95 cm) than in wild-type plants (118.14 ± 9.21 cm), with a decline of 42.82 and 47.42%, respectively (Figures 1B,C). However, plant heights were also decreased in *GhAPX1*-overexpressed cottons, with a decline of 19.17% in OA15 line and 18.46% in OA17 line (Figures 1B,C). The leaf size in *GhAPX1*-overexpressed cotton was not changed as compared with that in the wild-type plants. However, the leaf size was significantly decreased in IAO lines (Figure 1D). The leaf length was decreased in IAO24 line (14.10 ± 1.07 cm) and in IAO167 line (13.25 ± 0.63 cm) than in wild-type plants (20.06 ± 0.91 cm), with a decline of 36.08 and 39.94%, respectively (Figure 1E). The leaf width was also decreased in IAO24 line (2.36 ± 0.18 cm) and in IAO167 line (2.5 ± 0.08 cm) than in wild-type plants (3.39 ± 0.16 cm), with a decrease of 36.08 and 39.94%, respectively (Figure 1F).

### The Downregulation of Cytosolic Ascorbate Peroxidases Inhibited the Development of Sink Organs in Cotton

**FIGURE 2:**
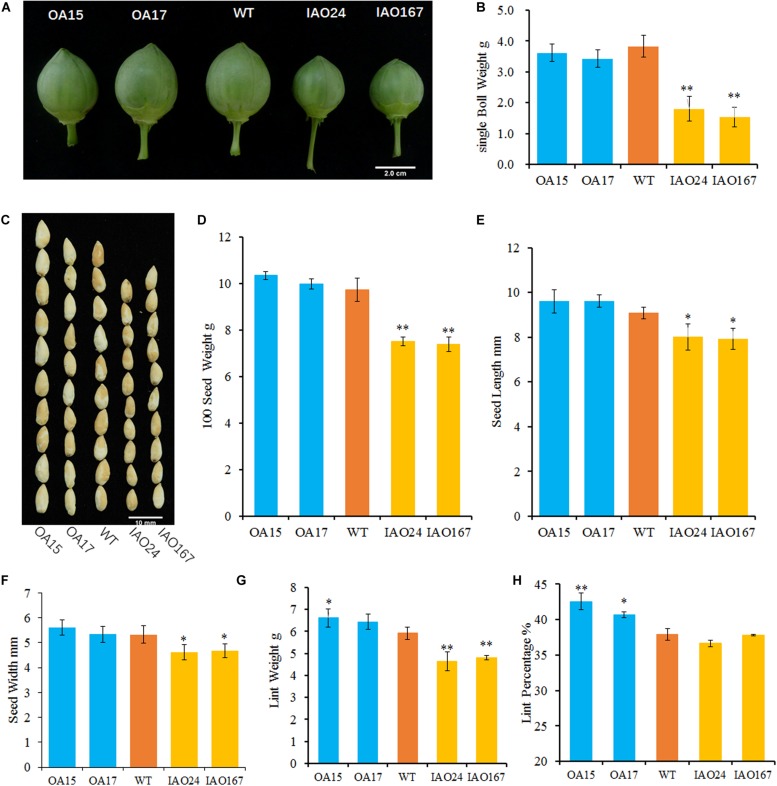
The downregulation of cytosolic ascorbate peroxidases (*APX*s) suppressed the development of cotton sink organs. **(A)** Cotton bolls at the stage of 30 days post anthesis (DPA) in wild-type plants and transgenic cottons. **(B)** The single boll weight of transgenic cottons compared with the control at mature stage. **(C)** The image of naked seeds in transgenic cotton. Bar = 10 mm. **(D)** The 100 seed weight of wild-type plants and transgenic cottons with three biological replicates (mean ± SD, *n* = 3). **(E)** The seed length of cytosolic *APX*-suppressed lines compared with the wild-type plant with three independent experiments (mean ± SD, *n* = 20 seeds of each line). **(F)** The seed width of cytosolic *APX*-suppressed lines compared with the wild-type plant with three independent experiments (mean ± SD, *n* = 20 seeds of each line). **(G)** The lint weight of the wild-type plants and transgenic cottons with three biological replicates (mean ± SD, *n* = 3). **(H)** The lint percentage of the wild-type plants and transgenic cottons with three biological replicates (mean ± SD, *n* = 3). ^∗^Indicates significant difference using Duncan’s multiple comparisons (^∗^*P* < 0.05; ^∗∗^*P* < 0.01).

To confirm whether the *cAPX*s could regulate the growth of sink organs, we checked the development of cotton bolls and fibers. The results showed that the change of *cAPX* expression levels alters the development of sink organs and affected cotton yield-related traits (Figure 2). The most obvious characteristic of phenotypic change is that the suppression of *cAPX*s inhibits the development of cotton bolls. The cotton bolls of IAO lines were smaller than the ones of wild-type at the stage of 30 DPA (Figure 2A). The single boll weight decreased from 3.83 ± 0.35 g in wild-type plants to 1.81 ± 0.39 g in IAO24 and 1.53 ± 0.32 g in IAO167, with a decrease of 52.84 and 60.10% at the mature stage (Figure 2B). However, the overexpression of *GhAPX1* did not increase cotton boll size and single boll weight at 30 DPA and mature stage (Figures 2A,B).

Yield-related traits including 100 seed weight and lint weight in IAO lines declined significantly compared with those in wild-type plants (Figures 2C–H). The 100 seed weight was decreased from 9.73 ± 0.49 g in wild-type plants to 7.49 ± 0.19 g in IAO24 and 7.38 ± 0.31 g in IAO167, with a decrease of 23.02 and 24.23%, respectively (Figures 2C,D). In the next step, we measured the seed size. The seed length was decreased in IAO24 line (7.98 ± 0.44 mm) and IAO167 line (8.03 ± 0.39 mm) than in wild-type plants (9.09 ± 0.35 mm) (Figures 2C,E). The seed width also decreased in IAO24 line (5.11 ± 0.32 mm) and IAO167 line (4.62 ± 0.32 mm) than in wild-type plants (5.33 ± 0.34 mm) (Figures 2C,F). The suppression of *cAPX*s inhibited the seed development. The effect of overexpressing *GhAPX1* on seed weight was slight and not significant (Figure 2D). Seed width was also not increased in overexpressed lines, and seed length had a small increase along with the upregulation of *GhAPX1* (Figures 2E,F).

Along with the change of *cAPX*s expression, the process of fiber development also changed (Figures 2G,H). Lint weights were increased in OA15 (6.62 ± 0.41 g) and OA17 (6.43 ± 0.35 g) lines than in wild-type cotton (5.92 ± 0.28 g). And there were a decrease of 23.02 and 24.23% in IAO24 (4.64 ± 0.43 g) and IAO167 (4.81 ± 0.11 g), respectively, than in wild-type plants (Figure 2G). Overexpression of *GhAPX1* increased lint percentage. The lint percentages were 42.54 ± 1.17% in OA15 line and 40.71 ± 0.39% in OA17 line, as compared with 37.92 ± 0.83% in wild-type plants, with an increase of 12.18 and 7.36%, respectively (Figure 2H). However, lint percentages were not changed in *cAPX*-suppressed lines IAO24 and IAO167 (Figure 2H). To further confirm the changes of fiber characteristic of transgenic cottons, we checked the number of fibers differentiated on the outer epidermis of the ovule collected at the morning of flowering day (0 DPA). The number of initiating fibers per unit surface area of overexpressed lines OA15 and lines OA17 was significantly higher than that of the wild-type plants. And the suppression of *cAPX*s had no effect on the initiation of fibers in IAO24 and IAO167 (Supplementary Figure 1). These results showed that the downregulated expression of *cAPX*s inhibited the development of cotton bolls, fibers, and seeds and reduced the storage capacity of sink organs.

### The Photosynthetic Rate Is Suppressed in Cytosolic Ascorbate Peroxidase Interfered Cotton

**FIGURE 3:**
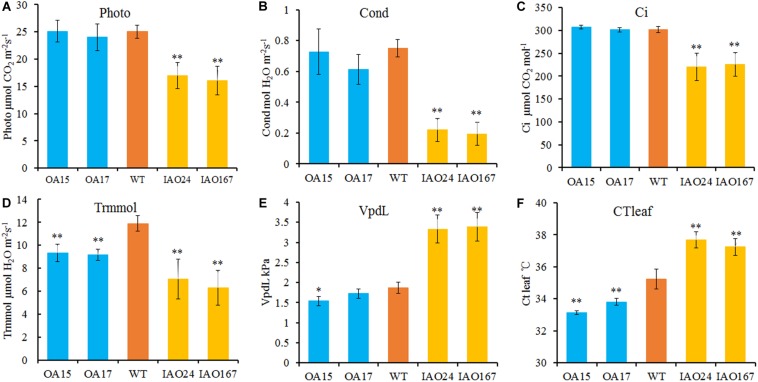
The photosynthetic rate is reduced in cytosolic ascorbate peroxidase (*APX*) interfered cotton. The main parameters of photosynthesis in overexpressed and cytosolic *APX*-suppressed cottons compared with wild-type plants. **(A)** Photo, net photosynthetic rate. **(B)** Cond, stomatal conductance. **(C)** Ci, intercellular carbon dioxide concentration. **(D)** Trmmol, transpiration rate. **(E)** VpdL, vapor pressure deficit. **(F)** CTleaf, leaf temperature. Data are means ± SD, *n* = 4 plants in each line at least three measurements. ^∗^Indicates significant difference using Duncan’s multiple comparisons (^∗^*P* < 0.05; ^∗∗^*P* < 0.01).

The decrease of plant development and yield-related traits is most likely due to the decrease of leaf photosynthetic ability. For H_2_O_2_, one substrate of APX is the important signaling molecule that regulates photosynthesis in plants (Gururani et al., 2015; Smirnoff and Arnaud, 2019). To determine whether changes in *cAPX* expression affected photosynthetic rate, we detected the photosynthetic rate of transgenic plants grown in field using a portable photosynthesis measurer. It showed that the suppression of *cAPX* expression significantly inhibited photosynthesis in cotton (Figure 3). The net photosynthetic rate (Photo), stomatal conductance (Cond), intercellular carbon dioxide concentration (Ci), and transpiration rate (Trmmol) were decreased significantly in IAO24 and IAO167 lines than in wild-type plants (Figures 3A–D), and steam pressure deficit on leaf surface (VpdL) and leaf surface temperature (CTleaf) rose sharply compared with those in wild-type plants (Figures 3E,F). However, overexpression of *GhAPX1* had little effect on the photosynthetic characteristics of the plants, except that the transpiration rate (Trmmol) and leaf surface temperature (CTleaf) were lower than in the control plant (Figures 3D,F). It means that *cAPX*s play a critical role in maintaining the photosynthetic rate in cotton leaf.

### Chlorophyll Contents Were Not Decreased in IAO Lines

There are many factors affecting the photosynthetic rate in leaves. Firstly, we measured the chlorophyll content in the third upper leaf. The contents of total chlorophyll and chlorophyll *a* were even increased slightly in IAO lines compared with wild-type plants. It meant that the decrease of photosynthetic rate was not due to the changes of chlorophyll content (Supplementary Figure 2). Then, we speculated that changes in the supply of carbon dioxide and water as raw materials for photosynthesis may be responsible for the decrease of photosynthesis rate in IAO lines.

### Reactive Oxygen Species Level Was Increased in Guard Cells of IAO Lines

**FIGURE 4:**
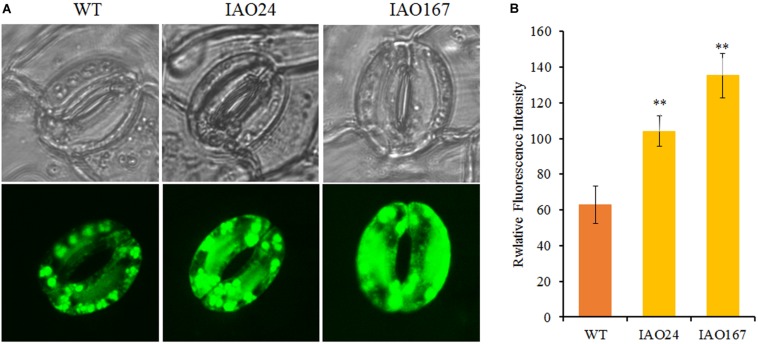
The downregulation of cytosolic ascorbate peroxidases (*APX*s) increased reactive oxygen species (ROS) levels in stomatal guard cells of abaxial leaf in cotton. **(A)** Qualitative detection of ROS in stomatal guard cells of cytosolic *APX*-suppressed lines and wild-type plants by staining with 10 μM of 2′,7′-dichlorodihydrofluorescein diacetate (2′,7′-DCFDA) and observing the fluorescent intensity with Laser TCS SP2 confocal spectral microscope, respectively. The upper panel is white light image; the bottom panel is fluorescent image. **(B)** The relative fluorescent intensity in stomatal guard cells of cytosolic *APX*-suppressed lines and wild-type plants. Data are means ± SD, *n* = 6 guard cells from three plants of each line. ^∗^Indicates significant differences using Duncan’s multiple comparisons (^∗∗^*P* < 0.01).

For intercellular carbon dioxide concentration (Ci), stomatal conductance (Cond) and transpiration rate (Trmmol) in the leaves of *cAPX*s interfered plants were decreased significantly (Figures 3B–D). The ROS level in stomatal guard cell is considered as the key factor to control photosynthesis, for it can regulate the stomatal pore size, opening, and closing (Song et al., 2014). *cAPX* is one of the major scavenging enzymes controlling H_2_O_2_ level in cell (Smirnoff and Arnaud, 2019). To confirm whether the suppression of *cAPX*s increased ROS level in guard cell, we detected relative ROS levels in guard cells in IAO lines with fluorescence dye 2′,7′-DCFDA (Figure 4). It showed that ROS fluorescence intensity in IAO lines were brighter than that in wild-type plants of guard cells (Figure 4A). The relative fluorescence intensity in IAO lines was also higher than that in wild-type plants (Figure 4B). And the stomatal opening in IAO lines was also smaller than that in wild-type plants (Figure 4A). These results suggested that the decrease of *cAPX* expression in leaves increased ROS level in stomatal guard cells, leading to the decrease of stomatal aperture, which might decrease the supply of carbon dioxide and water used for photosynthesis.

### The Downregulation of Cytosolic Ascorbate Peroxidases Increased Water Loss in Leaf

**FIGURE 5:**
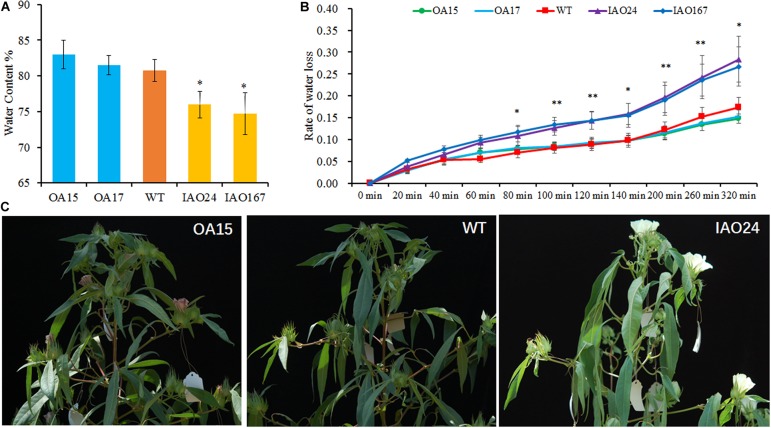
The downregulation of cytosolic ascorbate peroxidases (*APX*s) decreased the water content and increased the water loss rate in cotton leaf. **(A)** The water content was significant decreased in IAO lines compared with the wild-type plant. **(B)** The suppressed cytosolic *APX*s increased the water loss rate in leaf as compared with the wild-type plant. Data are means ± SD, *n* = 4 leaf samples from three plants in each line. ^∗^Indicates significant difference using Duncan’s multiple comparisons (^∗^*P* < 0.05; ^∗∗^*P* < 0.01). **(C)** The cytosolic *APX*-suppressed plants showed faster water loss and wilting symptoms in field at the middle of the day.

Water is one of the most important factors for photosynthesis. Therefore, we tested the water content of transgenic plants and found that the decreased expression of *cAPX*s resulted in a significant decline of water content in leaves (Figure 5A). The overexpression of *GhAPX1* could slightly increase the water retention rate of leaves (Figure 5A). We also tested the water loss rate of leaves. It was found that the water loss rate of *cAPX* plants was faster than that in wild-type and *GhAPX1*-overexpressed plant (Figure 5B). Moreover, we found that field-growing cotton loses water more quickly and is more prone to wilt at the middle of the day in summer at full bloom stage (Figure 5C). These data indicated that the downregulation of *cAPX* expression led to the decrease of water content and the increase of water loss rate in leaves, which caused the decrease of photosynthesis rate in IAO plants.

## Discussion

### Cytosolic Ascorbate Peroxidase Members Provide an Important Guarantee to Maintain Photosynthetic Rate

Stomatal pore is the main channel of carbon dioxide and water exchange between leaves and atmosphere. By adjusting stomatal size, the content of carbon dioxide and water in leaves can be regulated to control photosynthetic rate and stress tolerance of plants (Song et al., 2014). Guard cells can rapidly respond to changes in intracellular ROS level and make accurate responses to control stomatal behavior (Song et al., 2014). Thus, *cAPX* modulates REDOX balance of guard cells by controlling the content of hydrogen peroxide and reducing ASA in the cytoplasm, so as to control the degree of stomatal opening and closing, and finally regulates photosynthetic rate and the tolerance of plants to adversity (Pandey et al., 2017). In *Arabidopsis*, cytosolic *atapx1* mutants showed high sensitivity to the oxidative damage induced by MV, HL, and drought (Davletova et al., 2005; Suzuki et al., 2012). In rice, loss function of *OsAPX2* affected the growth and development in rice seedlings by protecting the seedlings from abiotic stresses (Zhang et al., 2013; Wu et al., 2018). The simultaneous interference of two *cAPX*s, *OsAPX1* and *OsAPX2*, could induce other peroxidases such as GPX and POX to maintain the stress tolerance (Bonifacio et al., 2011, 2016; Carvalho et al., 2014).

However, few studies explored the role of *cAPX*s in balancing plant stress tolerance and photosynthesis, although some studies have demonstrated that *cAPX* can regulate plant growth and development. In rice, the expression of one *APX* gene, *OsAPX1* or *OsAPX2*, was inhibited by RNAi; the plant height, leaf size, photosynthetic rate, and grain size were reduced (Rosa et al., 2010). T-DNA insert single mutation *osapx2* also caused serious suppression of plant development, the plant size became smaller and shorter, the leaves became smaller, and the fertility decreased significantly (Zhang et al., 2013; Wu et al., 2018). In *Arabidopsis*, *atapx1* mutant showed suppression of several crucial genes involved in basic plant growth and development, resulting in lower photosynthetic rates, slower growth, and delayed flowering under normal growth conditions (Pnueli et al., 2003; Davletova et al., 2005; Koussevitzky et al., 2008).

Recently, a study indicated that constant growth at high CO_2_ suppressed growth retardation in *atcat2* and *atapx1*/*atcat2* plants and lesion formation in *cat2* plants. It meant that plants were grown at high CO_2_ concentrations, a treatment that abolishes ROS production in peroxisomes (Vanderauwera et al., 2011). The CO_2_ concentration ([CO_2_]) in the atmosphere or leaves can regulate stomatal movement, stomatal development, stomatal conductance, and gas exchange between the leaves and the air to control plant development (Engineer et al., 2016). It indicates that there is a close relationship between the stomatal opening and the inhibition of plant growth caused by the deficiency of antioxidant enzyme in cells.

In this study, we found that increased ROS levels in stomatal guard cells of *cAPX*-suppressed cottons inhibited stomatal opening and decreased stomatal conductance and intercellular carbon dioxide concentration, which caused the decrease of photosynthetic rate (Figures 3, 4). The decrease of photosynthetic rate will directly lead to the decrease of photosynthetic products transported to sink organs (Supplementary Table 2). We examined the concentrations of glucose, fructose, and sucrose in fibers of transgenic cottons collected in the morning and afternoon. It showed that glucose, fructose, and sucrose concentrations were significantly decreased in fibers of IAO lines (Supplementary Table 2). These suggest that the suppression of in *cAPX*s inhibits photosynthesis and reduces the transport of photosynthetic products to sink organs, resulting in a decrease in yield (Figure 2 and Supplementary Table 2).

The decrease of stomatal aperture suppressed gas exchange between leaves and the atmosphere including H_2_O and CO_2_, which is the direct cause of the decline in photosynthetic rate (Figure 3). The low water content and fast water loss rate of leaves further confirmed the lack of water using for photosynthesis in cytosolic interfered lines (Figure 5). However, as a paradoxical result, the stomatal opening decreased, and the water loss rate should decrease, whereas the water loss rate increased in IAO lines in our study. This is mainly due to a number of factors that control the water loss rate in leaves, including stomatal opening, waxy thickness of leaf surface, and leaf epidermal cell arrangement (Zhu et al., 2010; Evans, 2013; Eva et al., 2019). The increase of VPD on the leaf surface and leaf surface temperature might be the one reason of increased water loss rate in IAO lines (Figures 3E, F). Stomatal opening is one of the many factors that control water loss. It may also be that changes in leaf structures and components accelerate water loss, but we were not sure that such factors were the reason of the faster water loss rate in leaves of IAO lines, for we did not check these in this study.

### Cytosolic Ascorbate Peroxidase Members Function Together to Control Plant Growth and Development by Regulating Reactive Oxygen Species Homeostasis

In this study, we found that the overexpression of *GhAPX1* did not increase photosynthetic rate in cotton and that the single boll weight did not increase too (Figures 2, 3). In a previous research, we also found that the specific suppression of *GhAPX1* in cotton, plant growth, and development, including leaf and seed size and fiber quality, were not changed significantly than in the control (Guo et al., 2016a). However, in the suppression of *cAPX*s including *GhAPX1*, *GhAPX8*, *GhAPX9*, and *GhAPX10*, the growth and development of plants were significantly inhibited, mainly manifested as the decrease of plant height, leaf size, photosynthetic rate, cotton boll, and seed size (Figures 1, 2). It suggests that *cAPX* family members have functional redundancy in cotton, which is different from that in rice and *Arabidopsis*.

Diploid rice and *Arabidopsis* have only two *cAPX* genes (Pandey et al., 2017). In the simultaneous interference of two *cAPX*s, *OsAPX1* and *OsAPX2*, there were no significant changes in leaf and seed, and the plant architecture was not significantly different from that of the wild-type (Bonifacio et al., 2011; Carvalho et al., 2014). Two *cAPXs* are not essential for photosynthesis in rice; alternatively, the deficient plants are able to trigger alternative oxidative and antioxidant mechanisms involving H_2_O_2_ signaling to maintain photosynthetic acclimation related to photochemistry, Calvin cycle, and photorespiration under ML and HL conditions (Carvalho et al., 2014; Bonifacio et al., 2016). However, the single mutation of *atapx1* or *atapx2* and double mutation of *atapx1* and *atapx2* all showed no obvious phenotypical changes in *Columbia-0* under normal condition (Suzuki et al., 2012).

A total of 26 *APX* genes were found in allotetraploid cotton, 12 of which were *cAPX* (Guo et al., 2016a; Tao et al., 2018). *GhAPX1* shows the highest similarity with *AtAPX1* and has the highest expression level among family members, and *GhAPX2* shows the highest similarity with *AtAPX2*. GhAPX8/9/10 is a new *cAPX* that is not found in rice and *Arabidopsis*, and *GhAPX8*/*9*/*10* appears to have a replication at the same locus on the chromosome (Guo et al., 2016a). These results indicate that the amplification of *cAPX* members plays a critical role in the plant development by maintaining the photosynthesis and stress tolerance in cotton. Thus, overexpression of a single APX gene does not have a significant effect on cotton growth and stress tolerance (Figure 2). A study showed that SCT plants that simultaneously overexpressed *GhSOD1* and *GhCAT1* appeared to benefit from synergistic effects of two genes and exhibited the highest tolerance to MV and salt stress, whereas the SAT plants simultaneously overexpressing *GhSOD1* and *GhAPX1* and a single overexpression of *GhAPX1* did not (Luo et al., 2013). In the study, even though the overexpressed *GhAPX1* gene increased the number of initiated fibers, lint weight, and lint percentage, there was no significant increase in single boll weight in overexpressed lines (Figure 2 and Supplementary Figure 1). The modification of a single *GhAPX1* gene expression has little effect on the photosynthetic rate, growth, and fiber quality of cotton (Guo et al., 2016a). However, the suppression of *cAPX*s would cause serious repression on photosynthesis and plant growth (Figure 2). Therefore, *cAPX* genes have functional redundancy in cotton to regulate growth and development. It requires specific promoters screening that used *cAPX* genes to improve plant growth or stress tolerance with genetic engineering method.

## Conclusion

The effects of ROS on photosynthesis rate are various. Most studies focus on the effect of ROS on the light and dark reactions during photosynthesis, including light energy absorption, electron transfer, photophosphorylation, carbon assimilation, and photorespiration (Gururani et al., 2015; Dietz et al., 2016; Smirnoff and Arnaud, 2019). There are few studies related to the effect of ROS scavenging enzymes on the absorption and utilization of carbon dioxide and water for plant photosynthetic. Based on the change of *cAPX* level, this study explores the role of ROS on photosynthesis and plant growth and development in cotton. The raised ROS levels in guard cells block the opening of stomatal pores, leading to a decline in plant photosynthesis, and eventually decrease the transfer of photosynthetic products, leading to a dramatic decrease of yield and quality in cotton.

## Data Availability Statement

All datasets generated for this study are included in the article/Supplementary Material.

## Author Contributions

LT and XZ conceived the original research plans and supervised and complemented the writing. KG, LT, and XZ designed the experiments. KG performed most of the experiments and analyzed the data. ZgL, HT, ZL, HH, PW, and ZY helped in the experiments. XD constructed the *GhAPX1*-overexpressed and *cAPX*-suppressed cottons. KG wrote the article with contributions of all the authors.

## Conflict of Interest

The authors declare that the research was conducted in the absence of any commercial or financial relationships that could be construed as a potential conflict of interest.

## Footnotes

1 http://www.plantgenetics.rug.ac.be/gateway/

## References

[B1] Apel K. Hirt H. (2004). Reactive oxygen species: metabolism, oxidative stress, and signal transduction. *Annu. Rev. Plant Biol.* 55 373–399. 10.1146/annurev.arplant.55.031903.141701 15377225

[B2] Begara-Morales J. C. Sanchez-Calvo B. Chaki M. Valderrama R. Mata-Perez C. Lopez-Jaramillo J. (2013). Dual regulation of cytosolic ascorbate peroxidase (APX) by tyrosine nitration and S-nitrosylation. *J. Exp. Bot.* 65 527–538. 10.1093/jxb/ert396 24288182 PMC3904709

[B3] Bonifacio A. Carvalho F. E. L. Martins M. O. Lima Neto M. C. Cunha J. R. Ribeiro C. W. (2016). Silenced rice in both cytosolic ascorbate peroxidases displays pre-acclimation to cope with oxidative stress induced by 3-aminotriazole-inhibited catalase. *J. Plant Physiol.* 201 17–27. 10.1016/j.jplph.2016.06.015 27379617

[B4] Bonifacio A. Martins M. O. Ribeiro C. W. Fontenele A. V. Carvalho F. E. Margis-Pinheiro M. (2011). Role of peroxidases in the compensation of cytosolic ascorbate peroxidase knockdown in rice plants under abiotic stress. *Plant Cell Environ.* 34 1705–1722. 10.1111/j.1365-3040.2011.02366.x 21631533

[B5] Carvalho F. E. Ribeiro C. W. Martins M. O. Bonifacio A. Staats C. C. Andrade C. M. (2014). Cytosolic APX knockdown rice plants sustain photosynthesis by regulation of protein expression related to photochemistry. Calvin cycle and photorespiration. *Physiol. Plant* 150 632–645. 10.1111/ppl.12143 24329817

[B6] Chang C. C. Ball L. Fryer M. J. Baker N. R. Karpinski S. Mullineaux P. M. (2004). Induction of ASCORBATE PEROXIDASE 2 expression in wounded *Arabidopsis* leaves does not involve known wound-signalling pathways but is associated with changes in photosynthesis. *Plant J.* 38 499–511. 10.1111/j.1365-313X.2004.02066.x 15086807

[B7] Considine M. J. Foyer C. H. (2014). Redox regulation of plant development. *Antioxid. Redox Signal.* 21 1305–1326. 10.1089/ars.2013.5665 24180689 PMC4158970

[B8] Davletova S. Rizhsky L. Liang H. Shengqiang Z. Oliver D. J. Coutu J. (2005). Cytosolic ascorbate peroxidase 1 is a central component of the reactive oxygen gene network of *Arabidopsis*. *Plant Cell* 17 268–281. 10.1105/tpc.104.026971 15608336 PMC544504

[B9] Dietz K. J. Turkan I. Krieger-Liszkay A. (2016). Redox- and reactive oxygen species-dependent signaling into and out of the photosynthesizing Chloroplast. *Plant Physiol*. 171 1541–1550. 10.1104/pp.16.00375 27255485 PMC4936569

[B10] Dong H. Bai L. Zhang Y. Zhang G. Z. Mao Y. Q. Min L. L. (2018). Modulation of guard cell turgor and drought tolerance by a peroxisomal acetate-malate shunt. *Mol. Plant* 11 1278–1291. 10.1016/j.molp.2018.07.008 30130577

[B11] Engineer C. B. Hashimoto-Sugimoto M. Negi J. Israelsson-Nordstrom M. Azoulay-Shemer T. Rappel W. J. (2016). CO2 sensing and CO2 regulation of stomatal conductance: advances and open questions. *Trends Plant Sci.* 21 16–30. 10.1016/j.tplants.2015.08.014 26482956 PMC4707055

[B12] Eva C. Oszvald M. Tamas L. (2019). Current and possible approaches for improving photosynthetic efficiency. *Plant Sci.* 280 433–440. 10.1016/j.plantsci.2018.11.010 30824023

[B13] Evans J. R. (2013). Improving photosynthesis. *Plant Physiol.* 162 1780–1793. 10.1104/pp.113.219006 23812345 PMC3729760

[B14] Foyer C. H. Noctor G. D. (2013). Redox signaling in plants. *Antioxid. Redox Signal.* 18 2087–2090. 10.1089/ars.2013.5278 23442120

[B15] Guo K. Du X. Tu L. Tang W. Wang P. Wang M. (2016a). Fibre elongation requires normal redox homeostasis modulated by cytosolic ascorbate peroxidase in cotton (*Gossypium hirsutum*). *J. Exp. Bot.* 67 3289–3301. 10.1093/jxb/erw146 27091877 PMC4892722

[B16] Guo K. Tu L. Wang P. Du X. Ye S. Luo M. (2016b). Ascorbate alleviates Fe deficiency-induced stress in cotton (*Gossypium hirsutum*) by modulating ABA levels. *Front. Plant Sci.* 7:1997. 10.3389/fpls.2016.01997 28101095 PMC5209387

[B17] Gururani M. A. Venkatesh J. Tran L. S. (2015). Regulation of photosynthesis during abiotic stress-Induced photoinhibition. *Mol. Plant* 8 1304–1320. 10.1016/j.molp.2015.05.005 25997389

[B18] Karpinski S. Escobar C. Karpinska B. Creissen G. Mullineaux P. M. (1997). Photosynthetic electron transport regulates the expression of cytosolic ascorbate peroxidase genes in Arabidopsis during excess light stress. *Plant Cell* 9 627–640. 10.1105/tpc.9.4.627 9144965 PMC156944

[B19] Koussevitzky S. Suzuki N. Huntington S. Armijo L. Sha W. Cortes D. (2008). Ascorbate peroxidase 1 plays a key role in the response of Arabidopsis thaliana to stress combination. *J. Biol. Chem.* 283 34197–34203. 10.1074/jbc.M806337200 18852264 PMC2590703

[B20] Lu Z. Liu D. Liu S. (2007). Two rice cytosolic ascorbate peroxidases differentially improve salt tolerance in transgenic *Arabidopsis*. *Plant Cell Rep.* 26 1909–1917. 10.1007/s00299-007-0395-7 17571267

[B21] Luo X. Wu J. Li Y. Nan Z. Guo X. Wang Y. (2013). Synergistic effects of GhSOD1 and GhCAT1 overexpression in cotton chloroplasts on enhancing tolerance to Methyl viologen and salt stresses. *PLoS One* 8:e54002. 10.1371/journal.pone.0054002 23335985 PMC3545958

[B22] Miao Y. Lv D. Wang P. Wang X. C. Chen J. Miao C. (2006). An Arabidopsis glutathione peroxidase functions as both a redox transducer and a scavenger in abscisic acid and drought stress responses. *Plant Cell* 18 2749–2766. 10.1105/tpc.106.044230 16998070 PMC1626619

[B23] Miller G. Suzuki N. Rizhsky L. Hegie A. Koussevitzky S. Mittler R. (2007). Double mutants deficient in cytosolic and thylakoid ascorbate peroxidase reveal a complex mode of interaction between reactive oxygen species, plant development, and response to abiotic stresses. *Plant Physiol.* 144 1777–1785. 10.1104/pp.107.101436 17556505 PMC1949877

[B24] Mittler R. Feng X. Cohen M. (1998). Post-transcriptional suppression of cytosolic ascorbate peroxidase expression during pathogen-induced programmed cell death in tobacco. *Plant Cell* 10 461–473. 10.1105/tpc.10.3.461 9501118 PMC144004

[B25] Nishiyama Y. Allakhverdiev S. I. Murata N. (2011). Protein synthesis is the primary target of reactive oxygen species in the photoinhibition of photosystem II. *Physiol. Plant* 142 35–46. 10.1111/j.1399-3054.2011.01457.x 21320129

[B26] Pandey S. Fartyal D. Agarwal A. Shukla T. James D. Kaul T. (2017). Abiotic stress tolerance in plants: myriad roles of ascorbate peroxidase. *Front. Plant Sci.* 8:581. 10.3389/fpls.2017.00581 28473838 PMC5397514

[B27] Pei Z.-M. Murata Y. Benning G. Thomine S. Klusener B. Allen G. J. (2000). Calcium channels activated by hydrogen peroxide mediate abscisic acid signalling in guard cells. *Nature* 406 731–734. 10.1038/35021067 10963598

[B28] Pnueli L. Liang H. Rozenberg M. Mittler R. (2003). Growth suppression, altered stomatal responses, and augmented induction of heat shock proteins in cytosolic ascorbate peroxidase (Apx1)-deficient *Arabidopsis* plants. *Plant J.* 34 187–203. 10.1046/j.1365-313x.2003.01715.x 12694594

[B29] Rizhsky L. Hallak-Herr E. Van Breusegem F. Rachmilevitch S. Barr J. E. Rodermel S. (2002). Double antisense plants lacking ascorbate peroxidase and catalase are less sensitive to oxidative stress than single antisense plants lacking ascorbate peroxidase or catalase. *Plant J.* 32 329–342. 10.1046/j.1365-313x.2002.01427.x 12410811

[B30] Rosa S. B. Caverzan A. Teixeira F. K. Lazzarotto F. Silveira J. A. Ferreira-Silva S. L. (2010). Cytosolic APx knockdown indicates an ambiguous redox responses in rice. *Phytochemistry* 71 548–558. 10.1016/j.phytochem.2010.01.003 20129631

[B31] Shen J. R. (2015). The structure of photosystem II and the mechanism of water oxidation in photosynthesis. *Annu. Rev. Plant Biol.* 66 23–48. 10.1146/annurev-arplant-050312-120129 25746448

[B32] Simkin A. J. Lopez-Calcagno P. E. Raines C. A. (2019). Feeding the world: improving photosynthetic efficiency for sustainable crop production. *J. Exp. Bot.* 70 1119–1140. 10.1093/jxb/ery445 30772919 PMC6395887

[B33] Smirnoff N. Arnaud D. (2019). Hydrogen peroxide metabolism and functions in plants. *New Phytol.* 221 1197–1214. 10.1111/nph.15488 30222198

[B34] Song Y. Miao Y. Song C. P. (2014). Behind the scenes: the roles of reactive oxygen species in guard cells. *New Phytol.* 201 1121–1140. 10.1111/nph.12565 24188383

[B35] Suzuki N. Miller G. Sejima H. Harper J. Mittler R. (2012). Enhanced seed production under prolonged heat stress conditions in *Arabidopsis thaliana* plants deficient in cytosolic ascorbate peroxidase 2. *J. Exp. Bot.* 64 253–263. 10.1093/jxb/ers335 23183257 PMC3528037

[B36] Tao C. Jin X. Zhu L. Xie Q. Wang X. Li H. (2018). Genome-wide investigation and expression profiling of APX gene family in *Gossypium hirsutum* provide new insights in redox homeostasis maintenance during different fiber development stages. *Mol. Genet. Genom.* 293 685–697. 10.1007/s00438-017-1413-2 29307114 PMC5948307

[B37] Tikkanen M. Grieco M. Aro E. M. (2011). Novel insights into plant light-harvesting complex II phosphorylation and ‘state transitions’. *Trends Plant Sci.* 16 126–131. 10.1016/j.tplants.2010.11.006 21183394

[B38] Vanderauwera S. Suzuki N. Miller G. van de Cotte B. Morsa S. Ravanat J. L. (2011). Extranuclear protection of chromosomal DNA from oxidative stress. *Proc. Natl. Acad. Sci. U.S.A.* 108 1711–1716. 10.1073/pnas.1018359108 21220338 PMC3029710

[B39] Wang P. Du Y. Li Y. Ren D. Song C. P. (2010). Hydrogen peroxide-mediated activation of MAP kinase 6 modulates nitric oxide biosynthesis and signal transduction in *Arabidopsis*. *Plant Cell* 22 2981–2998. 10.1105/tpc.109.072959 20870959 PMC2965546

[B40] Wang P. Song C. P. (2008). Guard-cell signalling for hydrogen peroxide and abscisic acid. *New Phytol.* 178 703–718. 10.1111/j.1469-8137.2008.02431.x 18373649

[B41] Wu B. Li L. Qiu T. Zhang X. Cui S. (2018). Cytosolic APX2 is a pleiotropic protein involved in H2O2 homeostasis, chloroplast protection, plant architecture and fertility maintenance. *Plant Cell Rep.* 37 833–848. 10.1007/s00299-018-2272-y 29549445

[B42] Yoshioka M. Uchida S. Mori H. Komayama K. Ohira S. Morita N. (2006). Quality control of photosystem II. Cleavage of reaction center D1 protein in spinach thylakoids by FtsH protease under moderate heat stress. *J. Biol. Chem.* 281 21660–21669. 10.1074/jbc.M602896200 16735503

[B43] Yoshioka-Nishimura M. Nanba D. Takaki T. Ohba C. Tsumura N. Morita N. (2014). Quality control of photosystem II: direct imaging of the changes in the thylakoid structure and distribution of FtsH proteases in spinach chloroplasts under light stress. *Plant Cell Physiol.* 55 1255–1265. 10.1093/pcp/pcu079 24891560

[B44] Zhang Z. Zhang Q. Wu J. Zheng X. Zheng S. Sun X. (2013). Gene knockout study reveals that cytosolic ascorbate peroxidase 2 (OsAPX2) plays a critical role in growth and reproduction in rice under drought, salt and cold stresses. *PLoS One* 8:e57472. 10.1371/journal.pone.0057472 23468992 PMC3585366

[B45] Zhu X. G. Long S. P. Ort D. R. (2010). Improving photosynthetic efficiency for greater yield. *Annu. Rev. Plant Biol.* 61 235–261. 10.1146/annurev-arplant-042809-112206 20192734

