# Supplementary material for: Cytosolic Ascorbate Peroxidases Plays a Critical Role in Photosynthesis by Modulating Reactive Oxygen Species Level in Stomatal Guard Cell
Source: Front Plant Sci. 2020 May 7;11:446. doi: 10.3389/fpls.2020.00446 (PMC7221183; doi:10.3389/fpls.2020.00446)
Supplement: Supplementary file 1 [file Presentation_1.pdf]

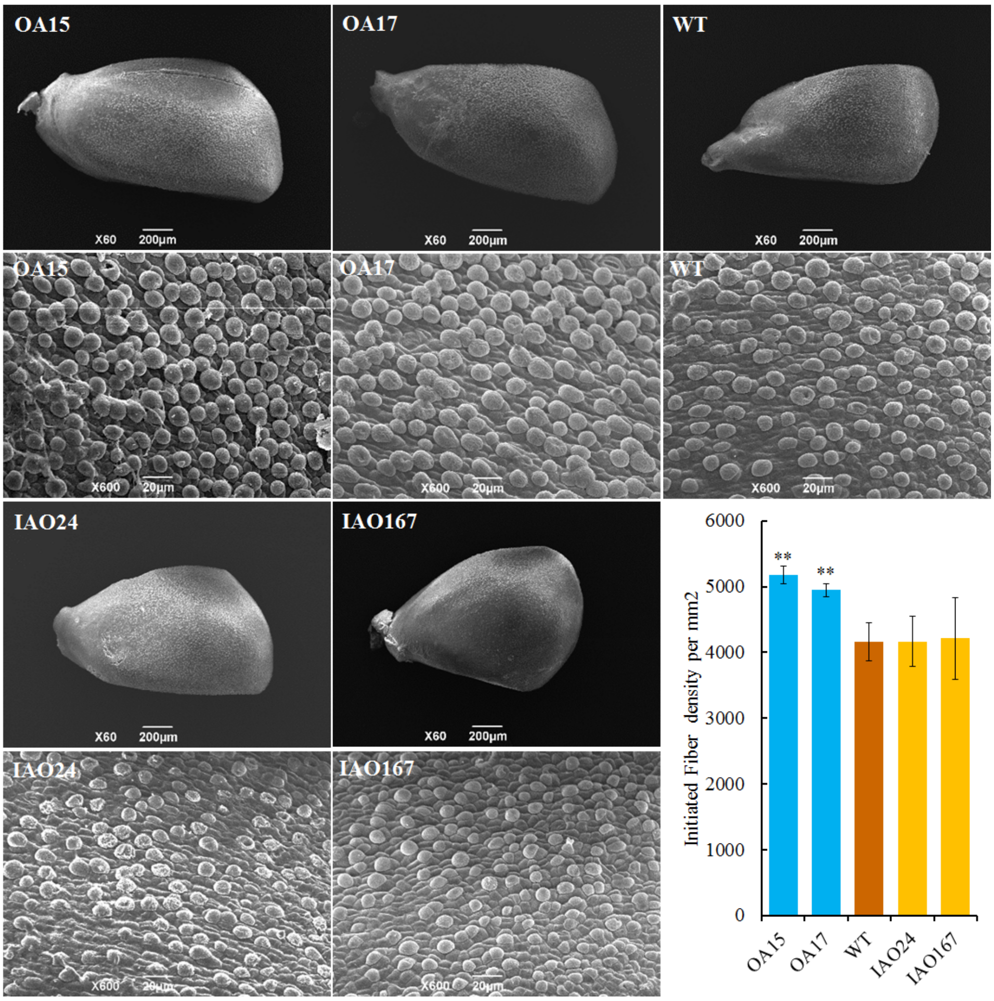

Supplementary Figure 1 The number of fibers initiated by cells on the outer epidermis of the ovule in the morning of flowering day (0 DPA) using scanning electron microscope (SEM) with. Datas are means ± sd, n = 3 ovules from 3 plants in each line. * indicates significant difference using Duncan's multiple comparisons, (*, $P < 0.05$; **, $P < 0.01$).

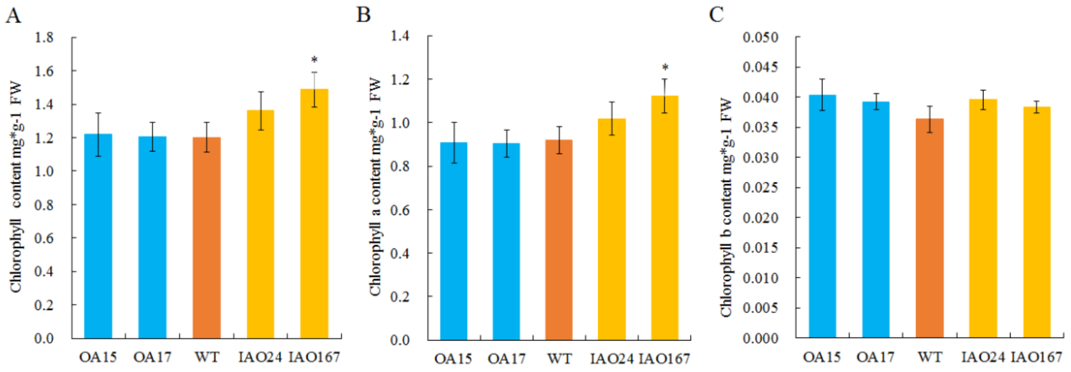

Supplementary Figure 2 The Chlorophyll content detection of transgenic cottons and wild plants. **(A)** the total chlorophyll content in different lines. **(B)** the chlorophyll a content in different lines. **(C)** the chlorophyll b content in different lines. Datas are means ± sd, 8 biological repeats. * indicates significant difference using Duncan's multiple comparisons, (*, $P < 0.05$;).
